# Out-of-pocket health expenditures as a barrier to healthcare access, financial protection, and progress toward universal health coverage: a narrative review from Somalia

**DOI:** 10.3389/fpubh.2026.1917671

**Published:** 2026-10-13

**Authors:** Sharmake Gaiye Bashir, Ilyas Abdullahi khalif, Mohamed Sharif Abdi, Yakub Burhan Abdullahi

**Affiliations:** Department of Public Health, Faculty of Medicine and Health Science, Hormuud University, Mogadishu, Somalia

**Keywords:** catastrophic health expenditure, health financing, out-of-pocket payments, Somalia, universal health coverage

## Abstract

Universal health coverage (UHC) requires both access to needed services and protection from financial hardship, yet Somalia's health system depends heavily on out-of-pocket (OOP) payments. This narrative review synthesizes recent evidence on the magnitude, drivers, and consequences of OOP spending in Somalia and identifies policy options to strengthen financial protection. OOP payments account for 44.9% of total health expenditure, far above the global (18.6%) and sub-Saharan African (35.7%) averages, while government financing and insurance coverage of about 3.5% remain minimal. Weak public financing, a dominant and largely unregulated private sector, and fragmented donor aid sustain this reliance. Roughly 24% of households experience catastrophic health expenditure, commonly resorting to borrowing, asset sales, and reduced consumption, and many forgo care altogether. These burdens fall disproportionately on the poor, women, rural populations, and internally displaced persons, leaving Somalia with the lowest UHC index among 120 countries at 33.5. Experiences from Rwanda, Ghana, and Ethiopia show that reducing OOP reliance depends on increased public financing, pooled prepayment, and pro-poor subsidies rather than insurance laws alone, supporting a phased, subsidy-backed reform agenda.

## 1 Background

Universal health coverage (UHC) is defined as ensuring that all people can access needed health services of sufficient quality without suffering financial hardship, linking service coverage and financial risk protection as core dimensions (1). Financial protection is most commonly undermined by out-of-pocket (OOP) expenditures, that is, direct payments by households at the point of service use, with no third-party payer (2). Globally, heavy reliance on OOP payments in low- and middle-income countries (LMICs) remains widespread and regressive, pushing over 800–927 million people into or deeper into poverty each year through catastrophic spending and medical impoverishment (1, 3). OOP shares above roughly 15–20% of total health expenditure are consistently associated with non-negligible levels of financial catastrophe and forgone care (1, 4). Evidence from LMICs shows that high OOP payment leads to catastrophic health expenditure, impoverishment, and widespread use of harmful coping strategies such as borrowing, selling assets, and cutting essential consumption, as well as forgone needed care (5, 6). Therefore, reducing OOP payments and expanding pooled, prepaid financing are central to UHC-oriented reforms and equitable access to care (4, 7).

Somalia's health system is one of the world's most fragile, with very low UHC index scores and severe deficits in infrastructure, workforce, and governance (8, 9). Health financing is dominated by donors and private spending, while the government accounts for less than 5% of total health sector expenditure, leaving households heavily exposed to direct payments and catastrophic costs (10, 11). OOP payments already constitute nearly 45% of total health expenditure, far above the global and regional averages, and health insurance coverage is extremely low (3). In this context, poor households face a dual burden: those who do seek care frequently incur catastrophic spending, while many others especially the poorest simply forgo needed services because they cannot afford them (3, 8).

Despite Somalia's extreme dependence on OOP financing and very low financial protection, existing analyses remain fragmented, often embedded within broader UHC or aid-dependency discussions rather than focused specifically on OOP dynamics and their consequences (8, 11). At the same time, survey evidence shows unusually high OOP shares, substantial catastrophic expenditure, and widespread impoverishing coping strategies, signaling that Somalia is an outlier even within sub-Saharan Africa and that OOP expenditure is a central barrier to UHC progress (1, 3).

The review's objectives are to: (i) describe the magnitude and patterns of OOP health spending in Somalia; (ii) assess its effects on healthcare access, catastrophic expenditure, impoverishment, and forgone care; (iii) analyze underlying health financing arrangements that sustain high OOP reliance; and (iv) identify context-appropriate policy options to reduce OOP payments and strengthen financial protection as core pathways toward UHC.

## 2 Methodology

### 2.1 Study design and rationale

This article was conducted as a narrative review. A narrative approach was chosen deliberately rather than a systematic review or meta-analysis because the evidence on out-of-pocket (OOP) health spending in Somalia is limited, fragmented, and methodologically heterogeneous. The available sources span very different designs, including household surveys, Demographic and Health Survey analyses, qualitative studies of internally displaced populations, national health-financing estimates, and gray literature produced by government bodies and development partners. Such diverse and non-comparable evidence cannot be pooled through the standardized extraction, quality-scoring, and statistical synthesis that a systematic review or meta-analysis requires. A narrative synthesis is better suited to integrating this body of work, interpreting findings across study types, situating Somalia within the broader sub-Saharan African and low- and middle-income country (LMIC) literature, and constructing a coherent conceptual account that links financing structures to healthcare access, financial protection, and progress toward universal health coverage (UHC). The narrative format also allows the review to draw transferable policy lessons from comparator countries, an analytical task that falls outside the scope of a purely quantitative synthesis.

### 2.2 Information sources and search strategy

Relevant literature was identified through structured searches of three bibliographic databases (PubMed, Scopus, and Web of Science), supplemented by Google Scholar to capture additional records and forward citations. Searches were conducted between **January** and **March 2026** and were restricted to sources published between 2021 and 2026. This window was chosen to reflect the post-2020 health-financing evidence base and to ensure that Somali expenditure estimates corresponded to the most recent national accounts and survey rounds. Search terms combined three concept blocks: financing concepts (“out-of-pocket,” “user fees,” “catastrophic health expenditure,” “financial protection,” “health financing,” “health insurance,” “risk pooling”); coverage concepts (“universal health coverage,” “healthcare access,” “forgone care”); and geographic terms (“Somalia,” “Somaliland,” “sub-Saharan Africa,” “fragile and conflict-affected settings,” “Rwanda,” “Ghana,” “Ethiopia”). Terms within each block were combined using OR, and the blocks were combined using AND. The reference lists of all included articles were hand-searched to identify further relevant studies.

Because Somalia's national health accounts are largely published outside the peer-reviewed literature, institutional and gray-literature sources were consulted in parallel to verify the financing figures reported in this review. These included the WHO Global Health Expenditure Database, World Bank data and Somalia health-sector assessments, and reports from the Federal Ministry of Health and its development partners. Each source was assessed for institutional provenance, transparency of method, and currency of data. Where the same estimates were also available in peer-reviewed syntheses, the peer-reviewed source was cited in preference, and the institutional source was used to confirm concordance. These sources are not counted among the records reported below.

### 2.3 Selection criteria and study selection

Sources were included if they addressed OOP expenditure, health financing, financial protection, healthcare access, or UHC, with direct relevance to Somalia or to comparable fragile, conflict-affected, or low-income settings. Both empirical studies and conceptual or policy-oriented analyses were considered, across quantitative, qualitative, and mixed-methods designs. Comparator-country evidence was included where it offered transferable insight into reducing OOP reliance. Rwanda, Ghana, and Ethiopia were purposively selected to represent three distinct financing pathways within sub-Saharan Africa: mandatory community-based insurance underpinned by strong state stewardship (Rwanda), a national scheme funded through a mix of taxation and contributions (Ghana), and incremental reform layering community-based insurance onto fee waivers and exemptions (Ethiopia). The selection was intended to span the range of prepayment models available to low-income African countries rather than to identify settings closely matched to Somalia. Sources were excluded if they lacked relevance to the review objectives, were not available in English, or did not contribute meaningfully to understanding OOP dynamics or financing reform. Selection was guided by relevance, methodological soundness, and contribution to the analytical narrative rather than by a formal eligibility checklist, consistent with the narrative-review methodology.

Database searches identified 170 records. After removal of 40 duplicates, 130 records were screened against the criteria above, of which 80 were excluded: 35 did not report OOP expenditure or financial protection outcomes, 25 were not relevant to the review topic, 15 did not contribute meaningfully to the review's thematic objectives, and 5 were not available in English. Fifty sources informed the final synthesis, of which 15 reported primary Somalia-specific evidence and 35 provided regional, comparator-country, or conceptual health-financing evidence. The selection process is summarized in Figure 1, and the characteristics and principal findings of the Somalia-specific sources are presented in Supplementary Table S1.

**Figure 1:**
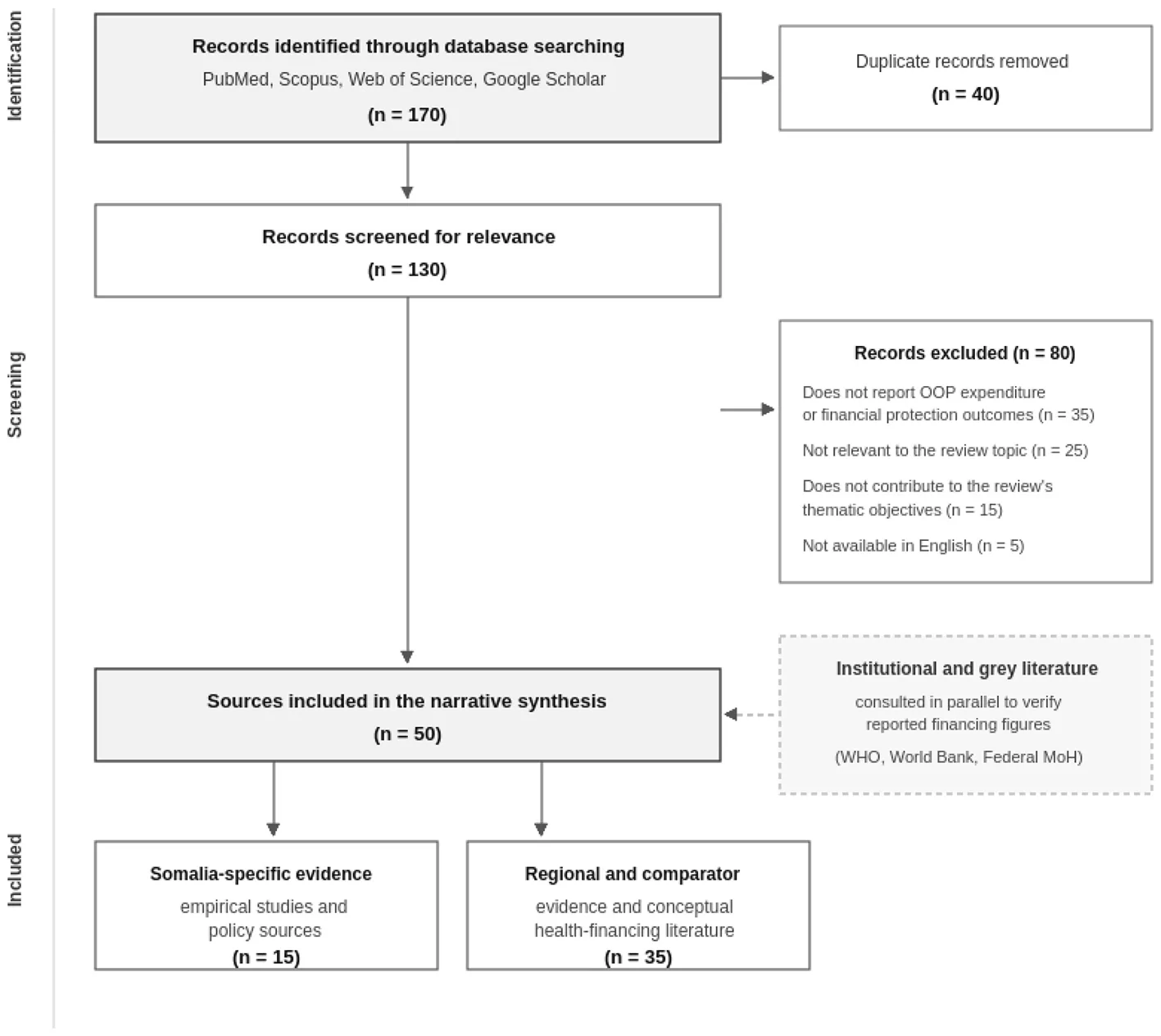
Flow of study identification, screening, and inclusion in the narrative synthesis.

### 2.4 Data synthesis

Evidence was synthesized thematically rather than statistically. Findings from the selected sources were extracted, grouped, and interpreted around the core themes of the review, namely the magnitude and components of OOP **expenditure**, its drivers, its consequences for access and financial protection, its implications for UHC and health-system performance, and the policy options available to reduce OOP dependence. Where relevant, quantitative indicators such as the OOP share of total health expenditure, the incidence of catastrophic health expenditure, and insurance coverage were reported descriptively and compared against global and regional benchmarks to contextualize Somalia's position. The experiences of Rwanda, Ghana, and Ethiopia were analyzed comparatively to identify reform mechanisms and lessons applicable to the Somali context. Throughout, evidence was organized to build a logical chain from financing structure to health and equity outcomes.

## 3 Overview of Somalia's health system and financing structure

### 3.1 Organization of the health system

#### 3.1.1 Public sector

The Federal Ministry of Health and federal member state ministries nominally lead the system but operate with severe capacity and fiscal constraints, in a context of fragile federalism and unclear roles across levels of government (9, 12). Public facilities cover only a fraction of primary care needs and depend heavily on donor-financed programs and NGOs (13).

#### 3.1.2 Private sector

Private providers dominate service delivery, providing approximately 80% of curative services and acting as the main referral hubs (11). This market-driven growth has occurred in the absence of strong regulation, producing wide variation in prices and quality and reinforcing OOP-funded, fee-for-service care.

#### 3.1.3 Development partners

Donors and UN agencies finance and implement a large share of EPHS and vertical programmes. While aid has expanded immunization and maternal–child health services, most funds flow off-budget through parallel supply and data chains, fragmenting governance and undermining state capacity and sustainability (11, 13).

This tripartite structure yields a de facto pluralistic system with weak stewardship, duplication, and gaps in coverage—conditions that systematically shift costs to households through OOP payments.

### 3.2 Sources of health financing

Somalia's health financing mix is dominated by household OOP payments and external aid, with marginal government contributions. Government health spending is among the lowest globally; one estimate finds health accounts for only 1.3% of total government expenditure, far below regional targets (10).

Donor funding, while substantial and high-performing in terms of disbursement (11), is highly fragmented and often tied to specific projects, creating misalignment with national priorities and limited financial protection functions (10).

Households fill the residual gap: external estimates suggest that OOP and donor funding each account for approximately 45% of total health expenditure (10). Diaspora remittances further finance private care and medicines (10). Their protective effect is real but unevenly distributed: remittances cushion recipient households while leaving those without diaspora connections fully exposed, and because they arrive as private income rather than pooled revenue, they carry none of the risk-sharing properties of prepayment. Their effect on measured hardship is therefore ambiguous, since remittances may reduce recorded catastrophic expenditure while masking the true extent of medical impoverishment, the cost of illness being transferred abroad rather than removed. Financial protection indicators for Somalia may consequently understate household exposure, and remittance-poor households, disproportionately the rural poor and the displaced, are those in greatest need of publicly financed protection.

### 3.3 Magnitude of OOP expenditure

Recent analysis estimates that OOP payments constitute 44.9% of Somalia's total health expenditure, drawn from WHO and World Bank data (3). This share far exceeds the global average of 18.6% and the sub-Saharan Africa average of 35.7%, positioning Somalia as an outlier in regional comparisons (3).

Household survey data show that 24% of Somali households face catastrophic health spending at the 10% income threshold, with widespread reliance on borrowing and asset sales, and many forgoing care due to costs (3). Among women in East Africa, Somalia records the highest proportion reporting money as a big problem in accessing treatment (64.8%), highlighting acute financial barriers (14).

From a normative perspective, the WHO and comparative UHC analyses suggest that OOP shares need to fall below approximately 15–20% of the total health expenditure for financial catastrophe to become negligible (1). Somalia's current OOP level is more than double this indicative threshold, underscoring a severe misalignment with global recommendations and the absence of effective financial risk pooling.

Taken together, the organizational fragmentation, minimal public spending, donor-driven verticalism, and dominance of an unregulated private sector structurally channel health system costs onto households, producing one of the heaviest OOP burdens in the region and severely constraining progress toward universal health coverage.

## 4 Understanding out-of-pocket expenditures in Somalia

### 4.1 Components of OOP spending

Household survey evidence from Somalia shows that out-of-pocket (OOP) spending spans nearly every stage of the care pathway. Consultation and treatment fees paid to private clinics and hospitals constitute a major cost component, reflecting the dominance of fee-charging private providers in service delivery (3). Medicines and medical supplies are the single largest driver of OOP burdens, as households frequently purchase drugs from private pharmacies even when attending public facilities due to recurrent stock-outs (3, 8). Diagnostic tests (laboratory and imaging) ordered by providers are another important contributor (3).

Hospitalization-related costs, including inpatient fees and ancillary charges, are not well quantified for Somalia but are major predictors of catastrophic expenditure in comparable settings such as Ethiopia and sub-Saharan Africa surgical care (6, 15). Transport and other non-medical costs are also substantial: Somali households, especially in rural areas, incur significant travel expenses to reach urban facilities, and indirect costs such as lost wages represent roughly half of total cholera episode costs (3, 16). Evidence from other African contexts confirms that transport, food, and lodging frequently precipitate catastrophic spending among chronic patients and surgical cases (17, 18).

### 4.2 Drivers of High OOP expenditure

Somalia's exceptionally high OOP share is sustained by intertwined demand- and supply-side determinants (3). On the supply side, extremely weak public financing only about 1.3% of government spending and roughly 5% of total health expenditure means that services and medicines are rarely fully funded from pooled resources (8, 10). Limited insurance coverage (approximately 3.5% of the population) leaves households effectively uninsured (10).

A fragmented system dominated by private and NGO providers, operating largely outside coherent purchasing and regulatory frameworks, is associated with user-fee-based care and unregulated prices for consultations, diagnostics, and drugs (10, 19). Governance weaknesses, including the absence of centralized drug regulation and fragmented donor funding, further exacerbate stock-outs and high medicine prices (8, 10).

Informal payments compound these formal costs. Systemic underfunding and weak governance have normalized off-the-record charges, with health workers facing salary precarity levying fees for nominally free services (20). The promise of free care at the point of service is therefore often illusory, and the most marginalized citizens, those the UHC framework is designed to protect, are priced out of the system by costs that appear nowhere in official schedules (20).

On the demand side, widespread poverty, informal employment, and low financial inclusion mean that even modest fees may trigger hardship, borrowing, asset sales, and forgone care; nearly two-thirds of Somali women report money for treatment as a “big problem,” with burdens concentrated among the poorest (3, 14). In such fragile settings, low measured catastrophic expenditure often reflects suppressed utilization rather than adequate protection, as many poor households simply do not seek care (8). Together, these factors help account for why OOP **expenditure** remains persistently high in Somalia.

## 5 OOP payments as a barrier to healthcare access

### 5.1 Delayed healthcare seeking

High OOP payment shape care-seeking decisions through anticipatory financial risk: households delay or downscale care when they expect unaffordable fees for consultations, tests, and medicines. In Somalia, survey data show that health expenses push many households into debt or asset sales, indicating that even necessary care is approached with caution and often postponed until illness is severe (3). Reviews from developing countries similarly document that OOP payments for malaria and other conditions lead to delayed treatment-seeking and reliance on self-medication or traditional healers, worsening outcomes and facilitating disease spread (21).

Among IDPs in Somaliland, OOP fees for registration, laboratory tests, and medicines are described as a “significant deterrent,” making affordability an “insurmountable wall” for many and lowering utilization even when services exist (22). This echoes broader evidence that cost barriers embed a cycle where poor health reduces earning capacity, further eroding ability to pay and encouraging late presentation (23).

### 5.2 Forgone care due to cost

Financial barriers frequently translate into complete non-use of services. In East Africa, nearly half of women report that “money needed for treatment” is a big problem; Somalia has the highest level (64.8%), with barriers concentrated among the poorest, least educated, and financially excluded women (14). National DHS analysis in Somalia similarly finds financial cost is the single most common barrier among married women (69.9%), exceeding distance, permission, or going alone (24).

In fragile contexts, this results in the - underuse of preventive and routine care. Somali IDP studies show low use of maternal and child health services, with financial constraints repeatedly cited alongside distance and insecurity as reasons for not attending ANC, skilled birth attendance, or postnatal care (25, 26).

At the system level, Somali households' heavy OOP burden (44.9% of total health expenditure) is reported to be “deterring utilization of needed services,” illustrating a direct macro-to-micro pathway from financing structure to forgone care (3).

### 5.3 Inequities in healthcare access

Financial barriers to care in East Africa are strongly pro-poor, with negative concentration indices indicating that the poorest shoulder the heaviest affordability problems (14). While simple comparisons often show rural disadvantage, multilevel analysis reveals that poverty, not rurality itself, drives these barriers; once wealth is controlled, rural–urban differences reverse, highlighting structural deprivation as the core mechanism (14).

In Somalia, married women in poorer wealth quintiles are far more likely to report barriers, and urban residents paradoxically report more barriers than nomadic populations, likely reflecting urban poverty and informal settlements where high living costs meet user-fee-based care (24).

Displacement compounds this gradient. Somali and Somaliland studies consistently show low utilization and high unmet need among internally displaced persons, with affordability and transport the dominant predictors of low service use (22, 27), consistent with global evidence that displaced populations experience worse access than other conflict-affected groups partly through financial exclusion (28).

Women and children bear the sharpest burden. Among married Somali women, over three-quarters report at least one barrier, with cost and distance most prevalent and large regional inequities (14, 24).

For children in Mogadishu IDP camps, vaccination uptake is undermined by opportunity costs and transport expenses even when vaccines are free, illustrating how non-fee costs still suppress utilization (29).

## 6 Financial and health consequences of OOP expenditure

Financial hardship from out-of-pocket (OOP) payments in Somalia manifests as catastrophic health expenditure, medical impoverishment, distress coping, and forgone care, with clear implications for health outcomes across disease areas. Somalia's experience is best understood within the broader theoretical and empirical work on financial risk protection in low- and middle-income countries (LMICs).

### 6.1 Catastrophic health expenditure and medical impoverishment

Catastrophic health expenditure (CHE) occurs when OOP payments exceed a defined share of income or consumption, forcing households to sacrifice basic needs, incur debt, or risk impoverishment (2, 30). In Somalia, 24% of households incur CHE at the 10% income threshold, more than double the global and African averages and well above the pooled sub-Saharan African incidence of approximately 16.5% (3).

Impoverishment follows when such spending pushes previously non-poor households below the poverty line or deepens existing poverty (2, 5). Evidence from Africa shows that even modest OOP burdens can tip low-income households into poverty (6, 31). In Somalia, where baseline poverty and informality are high, frequent CHE implies substantial though under-measured medical impoverishment (3).

### 6.2 Household coping mechanisms

In financial risk protection frameworks, “coping strategies” or hardship financing borrowing, selling assets, drawing down savings, or reducing consumption are a core indicator of financial distress (5). Somali data show that most households experiencing CHE resort to such strategies, including loans, asset liquidation, and cutting essential consumption (3).

Evidence from Ethiopia and other African settings corroborates that asset sales and borrowing are common responses to CHE and are strongly associated with long-term impoverishment and vulnerability (32, 33). Theoretically, such coping smooths short-term consumption but erodes productive capacity and resilience; reduced food and non-medical consumption directly harms nutrition and living standards, while indebtedness constrains future health-seeking and investment.

In Somalia, high rates of reported financial hardship and the use of distress strategies therefore likely mask a deeper cycle of vulnerability: households avoid immediate treatment gaps by borrowing or selling, but emerge poorer, less food-secure, and less able to absorb the next health shock (3).

### 6.3 Impact on health outcomes

Theoretically, OOP-induced hardship affects health via two pathways: (i) reduced or delayed use of needed care (forgone care for financial reasons), and (ii) deterioration of social determinants (nutrition, housing, education) when budgets are reallocated to health. The FRP literature explicitly links OOP burdens to forgone care and poorer health (5, 30).

In Somalia, high OOP payment is explicitly reported to deter the utilization of needed services, with many households postponing or forgoing care altogether (3). Regionally, women in Somalia report the highest financial barriers to seeking treatment in East Africa, implying significant adverse effects on maternal and reproductive health service use (14). Evidence from sub-Saharan Africa shows that CHE is frequently associated with hospitalizations, chronic illnesses, emergency surgeries, and maternal–child complications, with likely knock-on effects on morbidity and mortality (30, 32).

For communicable diseases, catastrophic and impoverishing spending for tuberculosis, HIV, and other infections is well documented in Africa and is associated with treatment interruption and poorer clinical outcomes (5, 34). For NCDs, chronic care requirements generate repeated OOP payment; scoping evidence from SSA finds very high CHE and near-universal unmet need for financial reasons among NCD patients, with extreme coping strategies common (35).

## 7 OOP expenditure, UHC, and health system performance

High OOP **expenditure** is associated with every dimension of universal health coverage in Somalia. Figure 2 sets out the proposed pathways linking Somalia's financing structure to its UHC outcomes, distinguishing households that forgo care from those that obtain it at catastrophic cost. Table 1 then details how OOP **expenditure** relates to financial protection, service coverage, and equity, and situates these dimension-specific patterns within the financing architecture that accompanies them.

**Figure 2:**
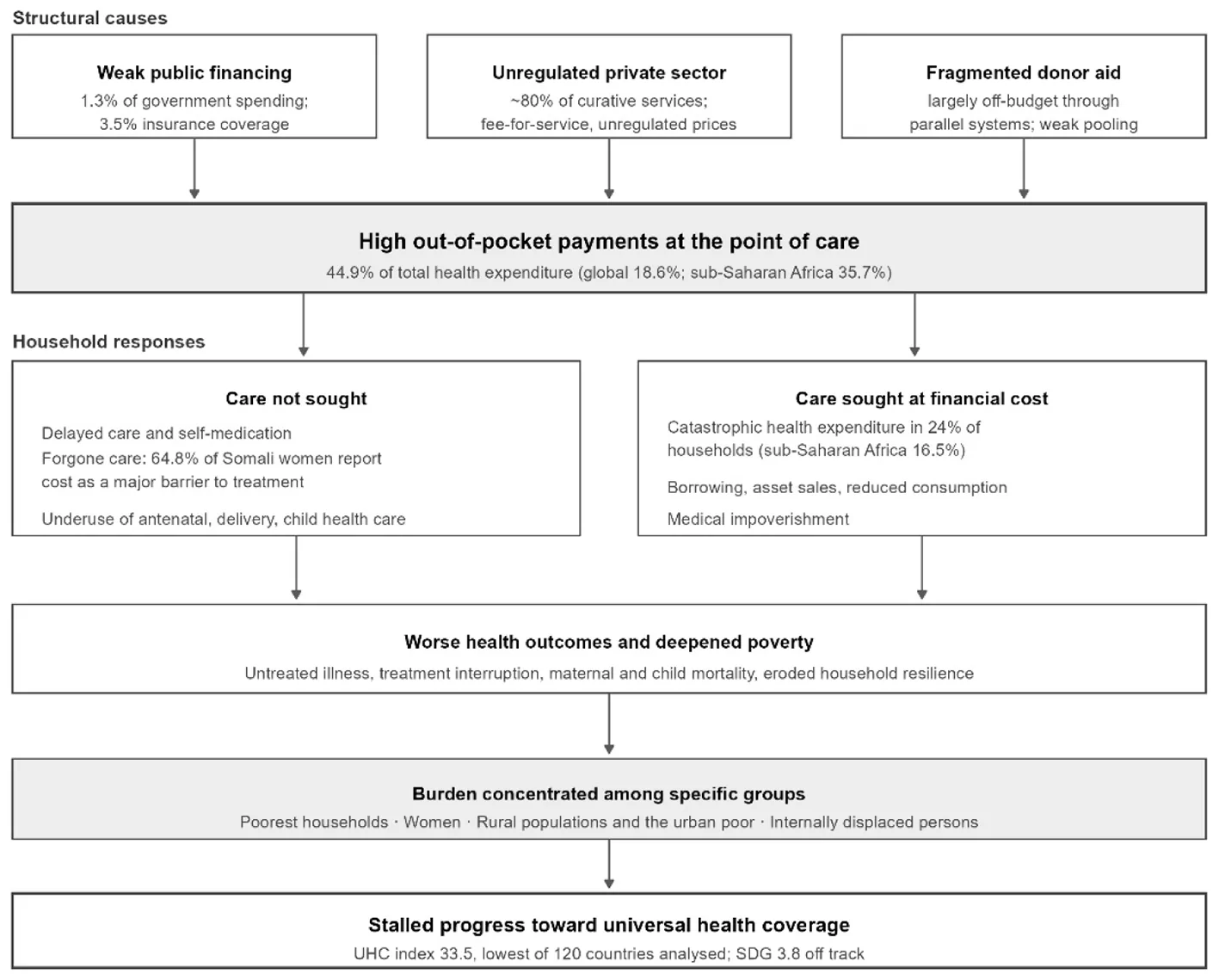
Conceptual framework of the pathways linking health financing structure to universal health coverage outcomes in Somalia. Weak public financing, an unregulated private sector, and fragmented off-budget donor aid are associated with a high share of health costs borne directly by households. High OOP payments are in turn associated with two household responses: care that is delayed or forgone, and care obtained at catastrophic financial cost. Both are likely to contribute to worse health outcomes and deepened poverty, with the burden concentrated among the poorest, women, rural populations, and internally displaced persons. Somalia has the lowest universal health coverage index among 120 countries analyzed. The arrows represent proposed relationships synthesized from observational evidence and do not denote established causal effects.

**Table 1:** High OOP expenditure and Somalia's progress toward universal health coverage: effects across UHC dimensions and overall trajectory.

UHC dimension	UHC objective	Effects of OOP spending	Consequences for health system performance	Implications for UHC achievement	References citations
Financial protection	Protect households from catastrophic and impoverishing health spending	OOP equals 44.9% of total health expenditure; 24% of households face catastrophic spending; most rely on borrowing, asset sales, or cutting essentials.	Increased impoverishment, instability of household consumption, and reduced resilience to shocks; high CHE signals a financing mix that fails to pool risk.	Persistent violation of the UHC financial protection goal; progress on SDG 3.8 stalled despite service delivery investments.	(3, 5, 8)
Service coverage	Ensure use of essential preventive and curative services	High expected OOP is associated with delayed care, self-treatment, and non-use of needed services, especially for women and poor households.	Underuse of preventive care, low coverage of ANC, skilled birth attendance, and child health interventions; high unmet need (about one-third of population).	Low national UHC index (33.5, lowest among 120 countries) reflects both gaps in service use and the financial barriers driving them.	(3, 8, 14)
Equity/population coverage	Reach vulnerable groups without discrimination	OOP payment are most burdensome for poorest, less educated, and financially excluded women; 64.8% of Somali women report money as a big problem.	Pro-rich inequity in access; socio-economically deprived groups are more likely to experience CHE and unmet need, indicating “system failure” where the poor do not seek care at all.	UHC pledge to “leave no one behind” is undermined; high OOP reproduces and amplifies existing socio-economic and gender inequities.	(8, 14)
Overall financing architecture and UHC trajectory	Mobilize sufficient pooled, prepaid revenue to sustain coverage	Government health spending is among the lowest globally and insurance covers only 3.5% of the population; donor aid flows largely off-budget through parallel systems, leaving households as the residual financier of care, medicines, and diagnostics even in public facilities	Absence of meaningful risk pooling and strategic purchasing; fragmented financing weakens stewardship and cross-subsidization	Without a substantial shift from direct payment to pooled public financing, Somalia is unlikely to meet SDG 3.8, and the UHC index will remain among the world's lowest	(1, 8, 11)

### 7.1 Synthesis of evidence

OOP expenditure in Somalia appears to function not as a financial inconvenience but as a systemic barrier associated with chronic fragility in the health system. The conceptual link between financing and outcomes rests on the absence of financial risk-pooling: without a mechanism to spread the cost of illness across the population, that cost remains an individual risk. Three patterns are apparent in the evidence reviewed. First, high OOP is associated with lower use of both preventive care and management of non-communicable diseases, and may contribute to higher morbidity. Second, health shocks are associated with movement of non-poor households into poverty and with deepening existing deprivation, as medical costs displace nutrition and education (3). Third, reliance on individual payments and fragmented donor aid is likely to limit the Ministry of Health's purchasing power and weaken stewardship (10, 20). Financial barriers therefore extend beyond formal fees, since even nominally free services carry a price, as described in Section 4.2.

## 8 International experiences and lessons for Somalia

As shown in Table 2, Rwanda, Ghana, and Ethiopia show that OOP reduction depends less on insurance laws alone than on how countries raise public revenue, pool funds, subsidize poor households, and enforce implementation. Somalia's main lesson is that prepayment can improve access and financial protection, but reforms transfer poorly when risk pools remain fragmented, public financing is weak, and service delivery capacity is low (1, 36).

**Table 2:** Health financing reforms and OOP reduction strategies in Rwanda, Ghana, and Ethiopia.

Country	Financing reform	Key features	Impact on OOP spending	Impact on healthcare access	Lessons for Somalia	References citations
Rwanda	National health insurance expansion centered on community-based insurance	Broad prepayment and risk pooling; strong political leadership; subsidies and exemptions to reduce hardship; Rwanda is among the few SSA countries with insurance coverage above 20%	Regional evidence associates Rwanda's robust CBHI and higher public spending with lower financial hardship; countries with coverage above 20% are characterized by substantial tax funding	Rwanda is associated with lower financial barriers and is repeatedly cited as a regional leader in progress toward UHC	Consistent with evidence from Rwanda and Tanzania, our results underscore the potential value of expanding social health insurance schemes or strengthened tax-funded systems.	(14, 37)
Ghana	National Health Insurance Scheme funded through mixed public revenue	Single system with a common benefit package funded by premiums, payroll tax, and earmarked VAT, with VAT providing 72% of funding	Ghana expanded coverage, but OOP remained important; by 2017 Ghana still classified as OOP-predominant, and insured households in West Africa still often incurred CHE	Ghana achieved one of the highest insurance coverage levels in SSA, but UHC progress remained incomplete and service coverage remained below 50% in one comparative review	Earmarked taxes can mobilize revenue in high-informality settings, but benefit-package promises must match provider payment, purchasing, and implementation capacity or informal and residual OOP persist	(39, 41)
Ethiopia	Incremental financing reform combining CBHI, fee waivers, exemptions, and facility financing reforms	Reforms included fee-waiver systems for the poor, exemption services, revenue retention, insurance, and facility autonomy; CBHI targeted rural and informal populations	Ethiopia still faces high OOP overall, but CBHI members had 28–43% lower annual OOP payments and lower catastrophic spending than non-members	CBHI increased outpatient use and improved financial protection, but national insurance coverage remained below one-third and uneven across groups and regions	Start pragmatically with subsidized schemes in stable areas, but note that voluntary CBHI is vulnerable to adverse selection, low renewal, and small fragmented pools, and remains dependent on sustained government subsidy; expand pooling over time, improve medicine availability and quality, and avoid leaving reforms at pilot scale	(38, 42, 47)

Before drawing lessons, the structural distance between these countries and Somalia should be stated plainly. Rwanda, Ghana, and Ethiopia each undertook reform from a position of functioning central administration, an established revenue base, and a public health system capable of delivering services at national scale. Somalia's government health expenditure remains among the lowest globally, administrative reach is uneven across federal member states, regulatory and enforcement capacity is limited, and much of the health system operates through private and non-governmental providers outside public financing arrangements (8, 10, 12). The comparator experiences are therefore treated here as sources of policy lessons about which mechanisms matter and under what conditions, rather than as models available for direct transfer. These three countries illustrate three distinct reform pathways: Rwanda relied on broad insurance expansion with strong state stewardship, Ghana on a mixed tax-insurance model with a national scheme, and Ethiopia on gradual reform that layered CBHI onto fee waivers, exemptions, and provider-side financing changes (37, 38). The common lesson is not that one institutional model fits all, but that lower OOP spending requires movement away from direct payment toward pooled prepayment, backed by public subsidies and implementation capacity (39, 40).

The contrast is especially important for Somalia because evidence across SSA shows that voluntary or fragmented insurance rarely achieves broad, equitable coverage in highly informal economies. Overall insurance coverage in SSA is low and pro-rich, and the few countries with coverage above 20% are all characterized by substantial tax funding (36). Cross-country panel analysis similarly finds that shifting from OOP-dominant financing to government-financed systems improves outcomes more than shifting to SHI-dominant systems, including a 3.3 percentage-point reduction in catastrophic expenditure incidence (41).

For Somalia, Rwanda's and Ghana's revenue strategies are more transferable than their institutional forms. Somalia shares with Ethiopia a large informal sector, weak fiscal space, and uneven administrative reach; therefore, Ethiopia's incremental approach is likely the most operationally realistic short-term model. Ethiopia's experience shows that CBHI can reduce OOP and increase service use, but also that coverage stalls when service quality, medicines, and administrative systems remain weak (42, 43). This warning is important in Somalia, where fragile systems, underfunding, and heavy OOP dependence produce the highest financial barriers in East Africa (14). The pitfalls of community-based insurance warrant explicit attention before it is adopted as a model. Because enrolment is voluntary and household-level, schemes tend to attract those who expect to use services while healthier households opt out, weakening the risk profile of already small pools (43). Renewal is frequently low, so coverage gains erode between enrolment rounds, and schemes remain dependent on continued government subsidy rather than becoming self-financing (42, 43). Fragmentation into many small district-level pools also limits cross-subsidization, the very function that makes prepayment protective (38). For Somalia, where administrative reach is uneven and fiscal space narrow, these risks are amplified: a CBHI pilot that enrolls the sick, renews poorly, and depends on donor subsidy would reproduce fragmentation rather than reduce it. The lesson is therefore conditional. CBHI is a defensible entry point only where premiums for poor households are fully subsidized from the outset, pools are designed for eventual consolidation rather than permanent district autonomy, and service readiness is sufficient that enrolment translates into actual care.

Somalia's main barriers to transfer are contextual in nature. Fragility limits revenue collection, regulation and enforcement. Fragmented financing impedes cross-subsidization, and highly segmented pools weaken solidarity (38, 39). The literature also warns that insurance on paper does not guarantee financial protection in practice: insured patients in SSA often still pay formal or informal OOP because of resource shortages, implementation gaps, and provider coping behavior (44). In addition, OOP is a structurally poor financing source in terms of efficiency, equity, sustainability, and feasibility, while external funding alone performs poorly in terms of equity and sustainability (4).

The main opportunities are equally evident. Somalia can begin with targeted pro-poor measures in relatively stable regions: fee waivers for priority services, donor-backed subsidies for poor households, and pilots of CBHI or other prepayment schemes where administrative control is feasible (14, 45). Over time, the stronger comparative lesson is to consolidate fragmented pools, increase the tax share of financing where possible, and adopt strategic purchasing so coverage expansion is matched by real provider payment and service availability (45).

International experience therefore supports a staged strategy for Somalia: immediate pro-poor financial protection, medium-term expansion of pooled prepayment in stable areas, and a longer-term transition toward more tax-funded and unified financing. Rwanda, Ghana, and Ethiopia do not offer a template, but together they show that reducing OOP requires political commitment, subsidized pooling, and implementation systems strong enough to turn entitlement into actual access.

## 9 Policy options for reducing OOP dependence in Somalia

Somalia can reduce out-of-pocket dependence through a phased package of higher public financing, tighter prioritization of essential services, targeted protection for poor households, and gradual pooling reforms rather than a rapid nationwide insurance rollout. The evidence favors feasible, subsidy-backed, and implementation-conscious reforms because Somalia's health system remains underfunded, fragmented, donor-dependent, and heavily reliant on private provision (3, 12). Table 3 sets out the main policy options, together with their mechanisms, expected benefits, feasibility, and key implementation challenges.

**Table 3:** Policy options for reducing reliance on OOP payments in Somalia.

Policy option	Mechanism	Expected benefits	Feasibility	Potential implementation challenges	Referenes citations
Increase government health expenditure	Raise domestic budget allocation and align donor support behind national priorities; expand fiscal space, since aid alone is not sustainable	Lower OOP, better financial protection, and improved UHC performance, as higher domestic public spending is associated with greater efficiency and a lower catastrophic burden	Moderate; achievable in phases if increases are incremental and tied to basic services and medicines	Very limited fiscal space and a weak revenue base; fragile public financial management; heavy donor dependence with much aid flowing off-budget; competing budget priorities	(3, 8, 48, 49)
Expand essential health service packages	Publicly finance a prioritized package of high-impact, cost-effective primary care and outreach services through progressive realization	Improves access to affordable basic care and concentrates scarce resources on services with largest UHC gains	High if limited initially to PHC, maternal-child care, and essential medicines	Supply shortages, weak infrastructure, and limited medicines regulation	(1, 3, 8)
Social health insurance	Start with formal-sector enrollment or pilots, with tax funding and subsidies for poor and informal groups rather than relying only on contributions	Can expand coverage and reduce OOP when backed by subsidies and clear benefits	Low nationally, moderate as pilot in stable areas	High informality, limited administrative capacity, and weak coverage of poor groups in African SHI experience	(3, 41, 45, 46)
Strengthen risk pooling mechanism	Merge or coordinate fragmented pools and move toward compulsory or tax-backed prepayment to enable cross-subsidization	Better equity, lower fragmentation, and stronger financial protection	Moderate long-term with legal and institutional reforms	Fragmented schemes, adverse selection, and weak institutions	(4, 38, 46)
Adopt strategic purchasing	Contract providers against defined outputs, use accreditation, and shift from passive funding to deliberate payment methods	Improves value for money, quality, and alignment of spending with EPHS priorities	Moderate if started with a few services and major providers	Weak contracting and accreditation capacity; limited provider performance data; dominance of unregulated private providers with little incentive to accept contractual terms	(3, 45, 48)
Protect vulnerable groups	Use fee waivers, vouchers, cash transfers, and premium subsidies for poor households, rural populations, women, and chronic illness groups	Immediate reduction in catastrophic spending and better equity in access	High if targeted geographically and by vulnerability	Targeting errors, fiscal sustainability, and weak data systems	(3, 8, 50)
Regulate the private sector	Set price, quality, and contracting rules for dominant private providers and drug markets; integrate them into public financing schemes	Curbs unregulated pricing of consultations, diagnostics, and medicines; reduces variation in service quality; brings the dominant private sector within public financing arrangements	Low to moderate in the short term; facility licensing and price transparency could begin in stable urban centers	Limited regulatory and enforcement capacity; absence of centralized drug regulation; provider resistance; risk of pushing services into informal markets	(3)

## 10 Recommendations

In the short term, the Ministry of Health and state health authorities should publicly finance a prioritized essential package, waive fees for high-priority maternal and child services, subsidize poor and rural households, and begin licensing and price regulation of private providers and medicine markets (3). These measures deliver immediate financial protection to those at highest risk of catastrophic spending (3, 8).

In the medium term, the Ministries of Finance and Health should phase in increases in domestic health spending tied to delivery of the essential package, and pilot subsidized community-based insurance in stable regions with premiums fully covered for the poorest households. Incremental pilots are more realistic than immediate national social health insurance where informality is high and administrative capacity weak (3, 39), provided each is paired with monitoring and service-readiness investment.

In the long term, government should consolidate fragmented funding into broader risk pools, strengthen strategic purchasing through contracting and accreditation of private providers, and shift the financing base toward general taxation. Sustained OOP reduction depends more on non-contributory government financing and merged pools than on fragmented contributory schemes (38, 41). The durable route is progressive universalism: expand a publicly financed core package, subsidize vulnerable groups first, and reduce OOP stepwise as capacity improves (3, 46).

Somalia's most realistic path is therefore a sequenced agenda: immediate pro-poor protection, medium-term pooling and spending reforms, and long-term unification of financing around UHC goals.

## 11 Study limitations

This review has several limitations inherent to its design. As a narrative rather than systematic review, it did not follow a registered protocol, apply a formal risk-of-bias or quality-appraisal tool, or use duplicate independent screening, which introduces a degree of subjectivity in source selection and interpretation. The Somalia-specific evidence base is thin, and several key figures derive from a small number of studies and modeled estimates, so some findings should be interpreted with caution. Reliance on gray literature and secondary financing estimates adds further uncertainty, and the restriction to English-language sources may have excluded relevant material. Finally, the heterogeneity of the underlying studies precluded quantitative pooling, meaning the synthesis is interpretive rather than statistical. Despite these constraints, the review provides a coherent and evidence-informed account of OOP spending in Somalia and a basis for policy and future research. Because most of the underlying evidence is observational and much of it cross-sectional, the relationships described in this review, including those depicted in Figure 2, should be interpreted as associations rather than established causal effects.

## 12 Conclusion

OOP payments are the defining weakness of Somalia's health financing system and a central obstacle to universal health coverage. At nearly 45% of total health expenditure, OOP reliance is associated with catastrophic spending in roughly a quarter of households and with widespread borrowing, asset depletion, impoverishment, and forgone care. Because there is almost no risk pooling, the cost of illness remains an individual burden that falls hardest on the poor, women, rural communities, and the displaced, precisely the groups UHC is meant to protect. These patterns are mutually reinforcing: high OOP is associated with suppressed utilization, deeper poverty, and weakened stewardship, and Somalia records the world's lowest UHC index. International experience suggests this trajectory is not inevitable, since countries that expanded public financing and pooled prepayment have seen reduced financial hardship and improved access. For Somalia, the realistic path is progressive universalism, starting with immediate pro-poor protection, advancing through medium-term pooling and spending reforms, and ending in long-term unification of fragmented financing. Without a deliberate, subsidy-backed shift away from direct payment, meaningful progress toward SDG 3.8 and financial protection is unlikely.
