# Supplementary material for: Out-of-pocket health expenditures as a barrier to healthcare access, financial protection, and progress toward universal health coverage: a narrative review from Somalia
Source: Front Public Health. 2026 Oct 13;14:1917671. doi: 10.3389/fpubh.2026.1917671 (PMC13711032; doi:10.3389/fpubh.2026.1917671)
Supplement: Supplementary file 1 [file Table_1.docx]

**Supplementary Table 1. Characteristics and principal findings of Somalia-specific sources included in the review**

| **Author (year) [ref]** | **Design** | **Sample** | **Setting** | **Outcomes assessed** | **Principal findings** |
| --- | --- | --- | --- | --- | --- |
| Mohamud et al. (2026) [3] | Cross-sectional household survey with regional and global benchmarking | 2,000 households (2023–24 national survey) | Somalia, national | OOP share of total health expenditure; insurance coverage; CHE incidence; coping strategies | OOP accounted for 44.9% of total health expenditure, against a global average of 18.6% and sub-Saharan African average of 35.7%; only 3.5% insured; 24% of households incurred CHE at the 10% income threshold; most resorted to borrowing, asset sales, or cutting essential consumption |
| Nikoloski et al. (2025) [8] | Secondary analysis of national survey data; composite UHC index construction, concentration-index equity analysis, logit modelling | Nationally representative household survey data | Somalia, national with regional disaggregation | Composite UHC index (service access and financial risk protection); equity in access; CHE risk | Somalia's UHC index of 33.5 was the lowest among countries previously analysed; socio-economic status and education were the dominant sources of disparity, favouring wealthier groups; the poorest faced the highest risk of catastrophic expenditure |
| Dirie et al. (2025) [9] | Short report; qualitative multi-stakeholder roundtable with thematic analysis | 30 purposively sampled participants (federal and state ministries, universities, providers, NGOs, civil society) | Mogadishu | Priority areas and implementation strategies for the 2025–2029 National Transformation Plan health pillar | Four priorities identified: primary health care expansion using female community health workers and rural facility rehabilitation; workforce development through regional training hubs (0.11 clinicians per 1,000); regulation via the National Health Professionals Council; and public-private and digital health partnerships. Implementation constrained by limited domestic financing and weak governance coordination |
| Ahmed et al. (2023) [10] | Policy analysis article (non-empirical), drawing on secondary data and case studies | Not applicable | Somalia, national | Health financing and governance obstacles to UHC | Infrastructure deficits, political instability, and limited domestic resources impede equitable coverage; aid and NGO provision fill gaps but are not sustainable; recommends strengthened governance, improved resource allocation, and innovative domestic financing |
| Hussein et al. (2026) [11] | Narrative review (1990–2024; final search April 2025); two reviewers, 221 records screened, 334 sources synthesised via thematic coding | 334 studies and reports | Somalia, with comparative cases | Aid effectiveness, sustainability, and governance consequences | Ministry of Health share of the national budget remained at or below 7%; donor inflows of approximately US$721.9 million flowed largely off-budget through vertical programmes and parallel supply and data chains, fragmenting governance and dampening state capacity; 2025 funding cuts curtailed essential services |
| Said and Kicha (2024) [12] | Perspective article | Not applicable | Somalia, national | Health governance and financing under federalism | Private providers dominate the sector while government health expenditure remains limited, leaving a substantial share of costs to households through OOP payments; per capita health expenditure is notably low and funding gaps are bridged through donor aid and development projects |
| Jama et al. (2023) [13] | Lessons-from-the-field implementation report | Stakeholder consultation over an 18-month revision process | Somalia, national | Revision of the essential package of health services | Public spending was estimated at 17% of total health expenditure and the UHC service coverage index at 27 of 100 against a regional average of 42.5; the revised package prioritised high-impact, cost-effective services under progressive realisation, with inclusivity and transparency identified as decisive to stakeholder alignment |
| Farih et al. (2026) [14] | Cross-sectional analysis of DHS data; concentration indices and multilevel logistic regression | 108,175 women (weighted) across eight East African countries, 2016–2022, including Somalia and Somaliland | East Africa | Self-reported "money needed for treatment" as a big problem | 49.7% of women across the region reported financial barriers, ranging from 64.8% in Somalia (the highest) to 36.2% in Tanzania; negative concentration indices in all countries confirmed pro-poor concentration; lower wealth, absence of formal education, and lack of a bank account predicted barriers, and the rural–urban disparity reversed after adjustment for socio-economic confounders |
| Figuereo et al. (2024) [16] | Cost-of-illness study | 400 patients and caregivers, plus facility staff, October–November 2023 | Five cholera treatment centres, Somalia | Household and facility costs per cholera episode; coping strategies | Mean household cost per episode was US$33.94, split almost evenly between direct (50.4%) and indirect (49.6%) costs; mean facility cost was US$82.65, giving a combined US$116.59 per episode; 61.8% of households drew on savings and 14.5% borrowed to meet costs |
| Korse et al. (2025) [21] | Community-based cross-sectional study; ordinal logistic regression | 271 households, proportionate stratified sample | Nasahablood and Ayah 4 IDP camps, Hargeisa, Somaliland | Level of healthcare utilization (low, moderate, high) | 73.5% of respondents reported low utilization and only 14.4% high; affordability was the strongest barrier (p<0.001), followed by physical accessibility (p=0.025), with both associated with substantially lower odds of service use |
| Ali et al. (2026) [23] | Cross-sectional multilevel analysis of the 2020 Somalia Demographic and Health Survey | 30,311 currently married women aged 15–49 | Somalia, national | Reporting at least one barrier to accessing healthcare (composite of four problems) | 77.06% reported at least one barrier; financial cost was the most common (69.91%), followed by distance (65.95%), reluctance to go alone (49.64%), and permission (46.03%); household wealth was strongly protective (richest vs poorest aOR 0.27) while urban residents reported more barriers than nomadic women (aOR 1.40); community-level context explained 26.3% of total variance |
| Mohamed et al. (2021) [24] | Qualitative study (in-depth interviews, focus groups, field observation) using the socio-ecological model | 17 in-depth interviews and 7 focus group discussions with mothers, providers, traditional birth attendants, and camp leaders | IDP settlements, Mogadishu | Barriers to maternal and child health service utilization | Barriers operated at every socio-ecological level: low socio-economic status and limited decision-making power individually; male dominance in decision-making and absent family support interpersonally; insecurity and distance at community level; and lack of privacy, transport costs, limited opening hours, and weak referral pathways organisationally |
| Dahie et al. (2025) [25] | Community-based cross-sectional study; logistic regression | 410 women who had given birth in the preceding six months, September–October 2023 | Ten IDP camps, Benadir region, Somalia | Prevalence and determinants of institutional delivery | Institutional delivery prevalence was 25%; antenatal attendance (AOR 13.30) and knowledge of danger signs (AOR 4.48) were the strongest positive predictors, while home delivery was driven by financial constraints, facility distance, lack of transport, and facility closures |
| Halane et al. (2026) [26] | Community-based cross-sectional study; multistage random sampling, logistic regression | 548 women of reproductive age (15–49) | IDP settlements, Banadir region, Somalia | Antenatal care, skilled birth attendance, and postnatal care utilization | Formal education predicted use of antenatal care (AOR 2.61), skilled birth attendance (AOR 3.46), and postnatal care (AOR 2.65); financial constraints and distance to facilities remained major barriers across all three services |
| Khalif et al. (2026) [35] | Commentary/perspective | Not applicable | Somalia, national | Informal payments and their equity consequences | Proposes the "illusion of free care," a four-stage process linking fee-exemption policy promises, chronic underfunding and workforce precarity, normalisation of off-the-record charges, and inequitable outcomes; informal payments are framed as predictable systemic responses rather than isolated corruption, disproportionately excluding the poorest and stratifying care by socio-economic status and gender |
| Jelle et al. (2023) [28] | Qualitative process evaluation nested within a cluster-randomised controlled trial of a participatory learning and action intervention; socio-ecological framework analysis | Observation data from 40 group discussions with female caregivers; 9 semi-structured interviews with health workers; 6 key informant interviews with policy influencers; review of 4 national vaccination policy documents; assessment of 5 health facilities | IDP camps, Afgooye Corridor, Kahda district, Mogadishu | Multilevel barriers to routine childhood vaccination uptake | Barriers operated at all five socio-ecological levels. Although vaccination is provided free by NGOs, opportunity costs for caregivers dependent on daily wage labour and transport costs for those living far from facilities suppressed uptake at the individual level, alongside limited knowledge and vaccine mistrust. Husbands and mothers-in-law constrained caregiver autonomy interpersonally; the Umul seclusion period and forced evictions to sites distant from facilities acted at community level; vaccine stock-outs (two of five assessed facilities were providing no vaccination services), long waiting times, language differences, and reluctance to open multi-dose vials operated organisationally; and confusion over eligible age and catch-up restrictions acted at policy level |

S1 The Somalia-specific evidence base is small, recent, and uneven. Of the 15 sources, four are non-empirical policy analyses, perspectives, or implementation reports, and only two report health expenditure directly at national scale; the remainder are cross-sectional or qualitative studies of service utilization, most conducted among internally displaced populations in Banadir and Mogadishu. No longitudinal or intervention study examines OOP expenditure in Somalia, and no source outside Mohamud et al. reports catastrophic expenditure incidence. This concentration of evidence in displaced urban populations, and the reliance of several key financing estimates on single sources, constrain the certainty of the synthesis and are reflected in the Study Limitations.
